# Negative-High Titer of *Helicobacter pylori* Antibody and Lipid Profiles

**DOI:** 10.1155/2022/9984255

**Published:** 2022-08-16

**Authors:** Jun Lu, Dong Van Hoang, Yuko Hayashi, Makiko Hashimoto, Sachiko Kubo, Hiroshi Kajio, Tetsuya Mizoue

**Affiliations:** ^1^Department of Medical Examination Center, Hospital of National Center for Global Health and Medicine, 1-21-1 Toyama, Shinjuku-Ku, Tokyo 162-8655, Japan; ^2^Department of Epidemiology and Prevention, Center for Clinical Sciences, Hospital of National Center for Global Health and Medicine, 1-21-1 Toyama, Shinjuku-Ku, Tokyo 162-8655, Japan

## Abstract

**Background:**

*Helicobacter pylori* (HP) is a causative factor for several gastrointestinal diseases. A HP seropositive antibody titer (i.e., ≥10 U/mL), a threshold indicating an HP infection, is known to be associated with changes in lipid metabolism. There is evidence that HP infection can be found in some individuals with HP antibody titer of between 3 and 9.9 U/mL (termed as “negative-high titer”). However, it is unknown about the relationship between HP negative-high titer and lipid metabolism. The present study aimed to quantify the association between HP negative-high antibody titer and lipid profiles.

**Materials and Methods:**

We surveyed 2,478 people who underwent a Ningen Dock examination and had serological HP antibody data, from May 2016 to December 2020 at National Center for Global Health and Medicine, Tokyo, Japan. Multiple regression models were used to quantify the association between HP antibody titer and serum lipid levels.

**Results:**

The adjusted odds ratio (95% confidence interval [CI]) for dyslipidemia in HP negative-high and positive titer was 1.24 (0.96, 1.79) and 1.36 (1.10, 1.68), respectively, compared with HP negative-low titer; *p* trend =0.005. The adjusted mean (95% CI) of high-density lipoprotein cholesterol (HDL-C) in HP negative-low, negative-high, and positive titer was 58.78 (57.86–59.71), 55.30 (53.70–56.91), and 53.76 (52.90–54.63) mg/dL, respectively; *p* trend <0.001. Higher HP antibody titers were also associated with higher ratio of low-density lipoprotein cholesterol (LDL-C) to HDL-C, but not triglycerides, or total cholesterols.

**Conclusion:**

The present cross-sectional study suggests that a HP negative-high antibody titer may be associated with dyslipidemia, HDL-C, and LDL-C to HDL-C ratio among Japanese Ningen Dock's participants.

## 1. Introduction

*Helicobacter pylori* (HP) is highly prevalent worldwide. It is estimated that more than half of the global population is infected with HP [1], and its prevalence is particularly high in East Asian countries, including China, South Korea, and Japan [2]. HP is a causative factor for a number of gastrointestinal diseases such as ulcerative or atrophic gastritis, and especially gastric cancer [3]. HP infection has also been linked to changes in the serum lipid profile, i.e., reduced high-density lipoprotein cholesterol (HDL-C) and triglycerides (TG), but elevated low-density lipoprotein cholesterol (LDL-C) and total cholesterol (TC) [4].

A serological test that combines anti-HP antibody with pepsinogen examination is a common tool for classifying HP infection into seronegative and seropositive HP status [5]. In Japan, serological HP antibody test kits, manufactured by Eiken Chemical Co. Ltd. (Tokyo, Japan), are commonly used. Accordingly, an anti-HP antibody titer of <10 U/mL is considered seronegative, and an anti-HP antibody titer of ≥10 U/mL is considered seropositive. However, there is evidence that some seronegative patients with an anti-HP antibody titer ranging between 3 and 9.9 U/mL have HP infection [6, 7]. Regarding the risk of HP infection and gastric cancer, it has been suggested that HP seronegative category should be further classified into “negative-high titer” (for those with anti-HP antibody titer between 3 and 9.9 U/mL) and “negative-low titer” (for those with anti-HP antibody titer of <3 U/mL) [8].

Although HP seropositive status is associated with changes in lipid metabolism compared with HP seronegative status [4], little is known about the lipid profile among those with HP negative-high titer compared with HP negative-low titer. Given the possibility of HP infection among those with HP negative-high titer [6, 7], we hypothesized that HP negative-high titer may be associated with dyslipidemia. To test this hypothesis, the present study investigated the relationship between HP antibody titer and lipid profiles.

## 2. Methods

### 2.1. Study Setting and Participants

In Japan, Ningen Dock is a special program which provides comprehensive health examination for early diagnosis and prevention of diseases, including measurement of blood lipids, and an optional test for HP infection. Participants of Ningen Dock program are required to complete a questionnaire to provide information on health-related lifestyle and medical history [9]. Eligible participants of the present study were those who participated in Ningen Dock program and underwent a serological HP antibody test. However, we excluded those with HP eradicated, who were using lipid-lowering medications, or who had missing information on lifestyle covariates.

**Figure 1:**
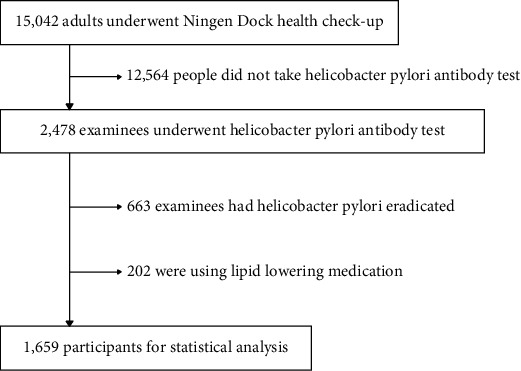
Participants and exclusion criteria

From May 2016 to December 2020, a total of 15,042 adult individuals came to the Department of Medical Examination Department, Center Hospital of the National Center for Global Health and Medicine (NCGM), Tokyo, Japan, for Ningen Dock health check-up. Of them, 2,478 people underwent a serological HP antibody test. However, we excluded 663 individuals who had HP eradicated, and 156 people who were using lipid-lowering medications, leaving an analytical sample of 1659 participants (Figure 1). The study protocol was approved by the Institutional Review Board for Clinical Research at the National Center for Global Health and Medicine (No. NCGM-G-003291-00). Information disclosure documents are posted on our hospital homepage.

### 2.2. Lipid Profile Assessment

Blood samples were collected in fasting status at the NCGM's Department of Medical Examination, by trained phlebotomist and nurses. Serum levels of TC, TG, HDL-C, and LDL-C were measured on an automatic biochemical analyzer (cobas c702 system; Roche Diagnostics International Ltd., Rotkreuz, Switzerland), under the monitor of well-trained inspectors. Dyslipidemia was defined as TG >150 mg/dL, LDL-C >140 mg/dL, or HDL-C <40 mg/dL [10].

### 2.3. HP Antibody

HP antibody data were measured with the LZ *H. pylori* antibody kit (380205-C; Eiken Chemical) on an automatic analyzer (7180 Clinical Analyzer; Hitachi High-Tech Corp., Tokyo, Japan). This measurement utilizes the latex agglutination reaction, and the change in turbidity caused by this reaction is measured optically to determine the antibody concentration. Following previous recommendation [7, 8], we categorized anti-HP antibody titer into three groups: “negative-low titer,” “negative-high titer,” and “positive titer,” for those with anti-HP antibody titer of <3, 3 to 9.9, and ≥10 U/mL, respectively.

### 2.4. Covariates

We also collected information on demographic characteristics, medical history, and lifestyle of participants, using the Ningen Dock's questionnaire [9]. Specifically, these covariates were age, sex, body height and weight (to calculate body mass index [BMI]), alcohol consumption (frequency and amount of alcohol drinking), sport activity (minutes per week), and smoking status.

### 2.5. Statistical Analyses

Participants' characteristics were summarized as mean ± standard deviation (SD) (for continuous variables) and percentages (for categorical variables). Differences in characteristics across categories of HP antibody titers (negative-low, negative-high, and positive) were examined using the chi-squared test (for categorical variables) and analysis of variance or the Kruskal-Wallis test (for continuous variables). The adjusted means and 95% confidence interval (CI) of individual lipid components (i.e., HDL-C, LDL-C, LDL-C to HDL-C ratio, and TC) across categories of HP antibody titer (negative-low, negative-high, and positive) were estimated from multiple linear regression models. TG was log-transformed to improve the normality before being fitted in linear regression models, and the mean and standard error were back-transformed. Logistic regression was used to estimate the odds ratio (OR) and 95% CI for dyslipidemia associated with HP antibody titers. The analyses comprised two models: Model 1, adjusted for age and sex (men or women); and Model 2, further adjusted for BMI (<18.5, 18.5–24.9, 25.0–29.9, or ≥30.0 kg/m^2^), alcohol consumption (0, <140, 140–280, or >280 g ethanol per week), physical activity (<60 or ≥60 min per week), and smoking status (non-smoker, ex-smoker, or smoker). Statistical significance was set at *p* < 0.05 (two-tailed). All statistical analyses were conducted using RStudio for Windows (version 3.2.4; RStudio Team 2012).

## 3. Results

Table 1 presents the participants' characteristics, stratified by HP antibody titer categories. Overall, the HP antibody titer seemed to have little relationship with BMI, sport activity, and alcohol consumption but was associated with age, sex, and smoking status. Participants with HP negative-high or positive antibody titer appeared to be older and were more likely women and current smokers, compared with those with HP negative-low antibody titer.

As presented in Table 2, higher titer of HP antibody was associated with higher odds of having dyslipidemia. The age- and sex-adjusted OR (95% CI) for dyslipidemia among those with HP negative-low titer (reference category), HP negative-high titer, and HP positive titer was 1.00 (reference), 1.31 (95% CI: 0.96, 1.79), and 1.36 (95% CI: 1.10, 1.68), respectively; *p* for linear trend was 0.005. The results remained almost unchanged after a further adjustment for BMI, alcohol consumption, physical activity, and smoking status. This pattern of association was similar after the negative-high titer being further divided into two subgroups: 3-6.4 and 6.5-9.9 U/mL (Table S1).

In Table 3, higher HP antibody titer was associated with a significantly lower serum level of HDL-C. The mean (95% CI) of HDL-C in HP negative-low, HP negative-high, and HP positive titer groups was 58.78 (57.86–59.71), 55.30 (53.70–56.91), and 53.76 (52.90–54.63) mg/dL, respectively (*p* trend <0.001). By contrast, a higher HP antibody titer was associated with a higher LDL-C to HDL-C ratio (*p* trend =0.017). HP antibody titer was not associated with LDL, TG, or TC. Further stratification of the lipid levels by two subgroups of negative-high titer (3-6.4 and 6.5-9.9 U/mL) also showed similar association pattern (Table S2).

## 4. Discussion

In the present cross-sectional study, a higher titer of HP antibody was associated with a significantly higher odd of dyslipidemia, higher ratio of LDL-C to HDL-C, but lower serum level of HDL-C, among adult people who underwent Ningen Dock health check-ups. HP antibody titer was not associated with the serum level of LDL-C, TG, or TC.

Our observed association between HP seropositive antibody titer and dyslipidemia is consistent with the up-to-date epidemiological data on the same topic. A recent meta-analysis of 27 case-control studies showed that HP seropositive individuals had higher serum level of TC (standardized mean difference [SMD] 0.09; 95% CI 0.07-0.10) and TG (SMD 0.06; 95% CI 0.05-0.08), but lower serum level of HDL-C (SMD -0.13; 95% CI -0.14 to -0.12), compared with HP seronegative ones [4]. In contrast, eradication of HP infection can lead to reduced risk of dyslipidemia (hazard ratio 0.85; 95% CI, 0.77–0.95) [11]. In the present study, the elevated odd of having dyslipidemia was even observed among those with negative-high titer compared with negative-low titer of HP antibody (OR 1.31; 95% CI 0.96, 1.79).

An elevated LDL-C to HDL-C ratio has been known as an important risk factor for coronary heart diseases [12, 13]. The present study showed that increasing HP antibody titer was associated significantly higher ratio of LDL-C to HDL-C, which, to the best of our knowledge, has not been investigated in the literature. The significant association was even observed when comparing HP negative-high v.s. HP negative-low titer. The present finding suggests that HP seronegative individuals should undergo serum lipid assessment, and that a HP serological test should be reported as not only negative/positive but also negative-gray values, i.e., between 3.0 and 9.9 U/mL.

One limitation of this study is that data were collected from a relatively small sample size of 2,478 examinees; thus, additional data should be collected. In addition, we did not determine the mechanism by which HP infection affects HDL-C. Meyer et al. [14] showed that HP follows a cholesterol gradient and extracts lipids from the plasma membranes of epithelial cells for subsequent glycosylation. Cholesterol glycosylation and extraction from host cells result in lipid raft destruction and alteration of the membrane architecture, which have been linked to immune evasion and bacterial persistence [14]. There may be a similar mechanism underlying the effect of HP infection on lipid profiles.

In conclusion, the present study suggests that a HP negative-high titer may be associated with a higher odd of dyslipidemia, a higher ratio of LDL-C to HDL-C, but a lower serum level of HDL-C.

## Figures and Tables

**Table 1:** Characteristics of *Helicobacter pylori*-negative and negative examinees.

	Overall	Helicobacter pylori antibody categories			*p* value
		Negative-low (<3 U/mL)	Negative-high (3-9.9 U/mL)	Positive (≥10 U/mL)	
*N*	1659	679	219	761	
Age (year), mean (SD)	47.5 (11.5)	47.1 (11.0)	45.2 (11.2)	48.7 (11.9)	<0.001
Sex (men), *n* (%)	972 (58.6)	422 (62.2)	115 (52.5)	435 (57.2)	0.023
Body mass index (kg/m^2^), *n* (%)					
<18.5	102 (6.1)	50 (7.4)	16 (7.3)	36 (4.7)	0.067
18.5–24.9	972 (58.6)	399 (58.8)	132 (60.3)	441 (58.0)	
25.0–29.9	500 (30.1)	187 (27.5)	63 (28.8)	250 (32.9)	
≥30.0	85 (5.1)	43 (6.3)	8 (3.7)	34 (4.5)	
Ethanol consumption (g/week), *n* (%)					
0	798 (48.1)	324 (47.7)	106 (48.4)	368 (48.4)	0.244
0 to <140	630 (38.0)	270 (39.8)	79 (36.1)	281 (36.9)	
140 to 280	118 (7.1)	48 (7.1)	21 (9.6)	49 (6.4)	
≥280	113 (6.8)	37 (5.4)	13 (5.9)	63 (8.3)	
Sport activity (min/week), *n* (%)					
<60	936 (56.4)	368 (54.2)	127 (58.0)	441 (58.0)	0.315
≥60	723 (43.6)	311 (45.8)	92 (42.0)	320 (42.0)	
Smoking status, *n* (%)					
Non-smoker	1009 (60.8)	400 (58.9)	129 (58.9)	480 (63.1)	0.018
Ex-smoker	288 (17.4)	142 (20.9)	34 (15.5)	112 (14.7)	
Smoker	362 (21.8)	137 (20.2)	56 (25.6)	169 (22.2)	

**Table 2:** Association between *Helicobacter pylori* antibody titers and dyslipidemia.

Regression model	Odds ratio (95% confidence interval) for dyslipidemia			P trend
	Negative-low <3 U/mL	Negative-high 3–9.9 U/mL	Positive (≥10 U/mL)	
*N* (%)	345 (50.8)	120 (54.8)	446 (58.6)	
Model 1	1.00 (ref)	1.31 (0.96, 1.79)	1.36 (1.10, 1.68)	0.005
Model 2	1.00 (ref)	1.24 (0.89, 1.71)	1.27 (1.02, 1.58)	0.035

Dyslipidemia was defined as triglyceride >150 mg/dL, low-density lipoprotein cholesterol >140 mg/dL, or high-density lipoprotein cholesterol <40 mg/dL; ref.: reference. Model 1: adjusted for age and sex; Model 2: further adjusted for body mass index, alcohol drinking, physical activity, and smoking status.

**Table 3:** Mean serum levels of lipid components across HP antibody titers.

Lipid components	Serum level of lipid (mg/dL), mean (95% CI)			*p* trend
	Negative-low <3 U/mL	Negative-high 3–9.9 U/mL	Positive (≥10 U/mL)	
*N*	679	219	761	
*HDL-C*				
Model 1	58.78 (57.86-59.71)	55.30 (53.70-56.91) ^∗∗^	53.76 (52.90-54.63) ^∗∗^	<0.001
Model 2	59.80 (58.49-61.12)	56.45 (54.64-58.26) ^∗∗^	55.24 (53.93-56.55) ^∗∗^	<0.001
*LDL*				
Model 1	125.99 (123.79-128.19)	126.30 (122.48-130.13)	128.97 (126.91-131.03)	0.89
Model 2	124.97 (121.67-128.28)	125.35 (120.81-129.89)	127.78 (124.49-131.06)	0.87
*LDL to HDL-C ratio*				
Model 1	2.27 (2.21-2.32)	2.41 (2.31-2.51) ^∗^	2.56 (2.50-2.61) ^∗∗^	0.017
Model 2	2.21 (2.13-2.30)	2.35 (2.23-2.47) ^∗^	2.48 (2.40-2.57) ^∗∗^	0.018
*Triglycerides*				
Model 1	98.28 (94.46-102.26)	102.10 (95.28-109.41)	104.89 (101.06-108.86) ^∗^	0.35
Model 2	106.54 (100.63-112.80)	108.58 (100.39-117.44)	110.58 (104.48-117.03)	0.63
*Total cholesterol*				
Model 1	201.39 (198.90-203.88)	197.10 (192.76-201.44)	198.44 (196.11-200.78)	0.09
Model 2	204.98 (201.23-208.73)	200.71 (195.56-205.86)	202.16 (198.43-205.89)	0.09

HP: *Helicobacter pylori*; HDL-C: high-density lipoprotein cholesterol; LDL: low-density lipoprotein cholesterol; CI: confidence interval; Model 1: adjusted for age and sex; Model 2: further adjusted for body mass index, alcohol drinking, physical activity, and smoking status; ^∗^*p* < 0.05; ^∗∗^*p* < 0.001.

## Data Availability

No data.

## Additional Points

*Significance Statement*. HP negative-high titer may be associated with dyslipidemia.

## Supplementary Materials

Supplementary Materials Table S1 showed that the negative-high titer group of Table 2 was further divided into two subgroups, 3-6.4 U/mL and 6.5-9.9 U/mL. In the same way, Table S2 stratified Table 3's negative-high titer part into two subgroups. The other analysis was the same as Tables 2 and 3, respectively.
