# Supplementary material for: Negative-High Titer of Helicobacter pylori Antibody and Lipid Profiles
Source: Biomed Res Int. 2022 Aug 16;2022:9984255. doi: 10.1155/2022/9984255 (PMC9398768; doi:10.1155/2022/9984255)
Supplement: Supplementary Materials — Table S1 showed that the negative-high titer group of Table 2 was further divided into two subgroups, 3-6.4 U/mL and 6.5-9.9 U/mL. In the same way, Table S2 stratified Table 3's negative-high titer part into two subgroups. The other analysis was the same as Tables 2 and 3, respectively. [file 9984255.f1.docx]

**Table S1: Association between *Helicobacter pylori* antibody titers and dyslipidemia**

| **Regression model** | **Odds ratio (95% confidence interval) for dyslipidemia** | | | | **P trend** |
| --- | --- | --- | --- | --- | --- |
|  | HP Negative-low titer (<3 U/mL) | HP Negative-high titer | | HP Positive titer  (≥10 U/mL) |  |
|  |  | (3–6.4 U/mL) | (6.5–9.9 U/mL) |  |  |
| N (%) | 345 (50.8) | 91 (53.5) | 29 (59.2) | 446 (58.6) |  |
| Model 1 | 1.00 (ref) | 1.20 (0.85, 1.70) | 1.77 (0.97, 3.27) | 1.35 (1.09, 1.68) | 0.005 |
| Model 2 | 1.00 (ref) | 1.12 (0.79, 1.60) | 1.77 (0.95, 3.37) | 1.27 (1.02, 1.58) | 0.029 |
| Dyslipidemia was defined as triglyceride > 150 mg/dL, low-density lipoprotein cholesterol > 140 mg/dL, or high-density lipoprotein cholesterol < 40 mg/dL; ref.: reference; Model 1: adjusted for age and sex; Model 2: further adjusted for body mass index, alcohol drinking, physical activity, and smoking status. | | | | | |

**Table S2: Mean serum levels of lipid components across HP antibody titers**

| **Lipid components** | **Serum level of lipid (mg/dL), mean (95% CI)** | | | | **P trend** | |
| --- | --- | --- | --- | --- | --- | --- |
|  | Negative-low  <3 U/mL | Negative-high | | Positive  (≥10 U/mL) | |  |
|  |  | 3–6.4 U/mL | 6.5–9.9 U/mL |  |  |  |
| N | 679 | 170 | 49 | 761 | |  |
| HDL |  |  |  |  | |  |
| Model 1 | 58.8 (57.9-59.7) | 55.1 (53.2-56.9) | 56.2 (52.8-59.6) | 53.8 (52.9-54.6) | | <0.001 |
| Model 2 | 59.8 (58.5-61.1) | 56.3 (54.3-58.3) | 56.9 (53.6-60.3) | 55.2 (53.9-56.5) | | <0.001 |
| LDL |  |  |  |  | |  |
| Model 1 | 126.0 (123.8-128.2) | 124.7 (120.4-129.1) | 131.8 (123.7-139.9) | 129.0 (126.9-131.0) | | 0.61 |
| Model 2 | 124.9 (121.6-128.2) | 123.5 (118.5-128.5) | 131.7 (123.2-140.1) | 127.7 (124.4-131.0) | | 0.56 |
| LDL to HDL ratio |  |  |  |  | |  |
| Model 1 | 2.27 (2.21-2.32) | 2.38 (2.26-2.49) | 2.53 (2.31-2.74) | 2.56 (2.50-2.61) | | 0.09 |
| Model 2 | 2.21 (2.13-2.30) | 2.31 (2.18-2.44) | 2.50 (2.28-2.72) | 2.48 (2.40-2.57) | | 0.14 |
| Triglycerides |  |  |  |  | |  |
| Model 1 | 98.3 (94.5-102.3) | 101.2 (93.6-109.5) | 105.3 (91.0-121.8) | 104.9 (101.0-108.9) | | 0.51 |
| Model 2 | 106.5 (100.6-112.8) | 107.3 (98.4-117.0) | 113.1 (97.8-130.8) | 110.5 (104.4-117.0) | | 0.86 |
| Total cholesterol |  |  |  |  | |  |
| Model 1 | 201.4 (198.9-203.9) | 195.3 (190.4-200.2) | 203.3 (194.1-212.5) | 198.4 (196.1-200.8) | | 0.031 |
| Model 2 | 204.9 (201.2-208.7) | 198.9 (193.2-204.5) | 207.1 (197.5-216.6) | 202.1 (198.4-205.8) | | 0.030 |
| HP: *Helicobacter pylori*; HDL: high-density lipoprotein cholesterol; LDL: low-density lipoprotein cholesterol; CI: confidence interval; Model 1: adjusted for age and sex; Model 2: further adjusted for body mass index, alcohol drinking, physical activity, and smoking status; * P < 0.05; ** P < 0.001 | | | | | | |
